# Choosing and enjoying violence in narratives

**DOI:** 10.1371/journal.pone.0226503

**Published:** 2019-12-19

**Authors:** Victoria Lagrange, Benjamin Hiskes, Claire Woodward, Binyan Li, Fritz Breithaupt

**Affiliations:** 1 Department of French and Italian, Indiana University, Bloomington, Indiana, United States of America; 2 Department of English, Indiana University, Bloomington, Indiana, United States of America; 3 Department of Germanic Studies, Indiana University, Bloomington, Indiana, United States of America; 4 Department of Cognitive Science, Indiana University, Bloomington, Indiana, United States of America; Trinity College Dublin, IRELAND

## Abstract

We use an interactive story design in which participants read short stories and make two consecutive plot choices about whether protagonists commit low- or high-violence actions. Our study has four main findings. 1) People who choose high violence report greater satisfaction with the story, while those switching to or staying with no violence show lower satisfaction. 2) However, when participants encounter these stories without choices, they reliably rate higher-violence stories as less satisfying than lower-violence stories. 3) Regret seems to account for the low satisfaction of those who choose or switch to low violence. 4) There is a large segment of people (up to 66%) who can be persuaded by different story contexts (genre, perspective) to choose extreme violence in interactive fiction and as a consequence of their choice feel satisfaction. We hypothesize that people who opt for high violence enjoy the story as a result of their choice. Overall, we suggest that choosing violence serves as a gateway for enjoyment by creating an aesthetic zone of control detached from morality.

## Introduction

Depictions of violence are highly present in many interactive media, and a matter of great controversy. Some media users show an interest in violence and derive high enjoyment from it. At the same time, other media users do not enjoy but rather detest and avoid violence. However, there also seem to be some people in the middle who sometimes, but not always, enjoy and choose violence in media. We are interested to know more about this middle group, such as how large it is and under what circumstances they will opt for and enjoy violence in media. Moreover, we are curious about the connection between choosing violence and enjoying it.

There have been mixed findings as to whether violence in media increases or decreases enjoyment. Some studies report increased enjoyment [1–2], some decreased enjoyment [3], and some no effect of self-reported enjoyment [4]. We add to this discussion specific narrative factors that can influence enjoyment of violence. We also add the finding that there is a surprisingly large proportion of the population that these factors can influence to opt for high violence in narratives with high satisfaction. Moreover, we provide a potential explanation for how violence in interactive media produces satisfaction. This set of studies is thus a contribution to the study of media and media psychology since it offers insights into when violence in media is enjoyable and when not. It is also a contribution to empirical narratology to demonstrate the value of interactive fiction to test successful plot-structures.

While many previous studies have focused on visual media, we focus on interactive fiction where readers choose how the plot should continue. Participatory or interactive fiction [5], a medium that has gained prominence with the internet, is an understudied genre [5–6] of growing importance, with movies such as Netflix’s *Black Mirror Bandernsnatch*, video games like Quantic Dreams’ *Heavy Rain*, *Beyond*: *Two Souls* or Dontnod Entertainment’s *Life is Strange*, online role playing games (RPGs), and hypertext fiction attracting increasing numbers of participants.

Interactive fiction reduces the complexity of other media, such as video games, by eliminating the visual aspects and demands of immediate reaction, and allows focus on plot choices. By concentrating on interactive fiction, we want to draw more attention to this genre. Studies of interactive fiction might also provide insights that could apply to video games and narrative fiction in general. While the choice making process in interactive fiction is different from video games and narrative fiction, some similarities remain. This is obvious in video games where users also make choices, though they may be under time pressure to react and perceive the choices paired with visual stimuli. But it additionally applies to narrative fiction where readers and listeners often identify with the protagonist and thus co-experience the decision-making with the protagonist [7–8].

The design of our studies uses interactive fiction by offering readers two consecutive plot decisions that contain varying degrees of violence. We ask our participants at different points of the study to rate their enjoyment with the narrative. Our studies use an understanding of violence as physical aggression in line with Anderson and Bushman: “violence refers to extreme forms of aggression, such as physical assault and murder” and aggression to “a behavior intended to harm another individual who is motivated to avoid that harm” [9]. For this and also all terms of this study, see Table 1.

**Table 1:** Key terms of our study.

Fiction	We understand fiction as narratives that contain imaginary actions and people. We consider fiction as “make-believe” [10] in any media, and do not restrict it to literature.
**Interactive or participatory fiction**	Interactive or participatory fiction is a narrative genre of fiction that offers choices for the continuation of a story [5]. It exists in printed form, but is more common on the internet.
**Genre**	We use genres of fantasy, realistic-contemporary, general historical settings, and a stylized Nazi-historical story with the same basic plot.
**Perspective**	We use second and third-person perspective. Stories are either presented using the “you” form or “she/he,” respectively
**Choice**	Choice refers to the presentation of different possible plot paths that continue the story. Participants can select which one path to take, but cannot go back and select a different one. (Also see Agency).
**Violence**	We follow Anderson & Bushman’s description of violence: “Violence refers to extreme forms of aggression, such as physical assault and murder” [9]
**Aggression**	We understand aggression as “a behavior intended to harm another individual who is motivated to avoid that harm” [9]
**Regret**	In the context of our studies, we understand regret as the wish to correct a previous aggressive choice by opting for an apology in future choices. We use the term regret as a moral emotion in line with Bell [11]: “the individual will appear, after the fact, to have made the wrong decision, even if in advance, the decision appeared correct with the information available at the time” [11]
**Satisfaction/ enjoyment**	Satisfaction is understood as enjoyment with the overall fiction. We do not distinguish between the two in this study.
**Agency**	We define agency as the feeling of control rather than actual control itself.
**Degrees in violence**	We study three degrees in violence: - No violence, which appears as non-confrontational communication. - Low violence, which can be defined as a physical aggression that does not threaten the life of the individual. - Extreme violence, that we understand as a physical aggression that threatens the life of the individual.
**Middle group**	We refer to the middle group of participants as those people who might opt for a choice of high violence in some story condition, but for no or low violence in others.

Overall, we ask:

1 What are the genre and perspective conditions under which people are more likely to opt for highly violent plot developments?

While a large part of the population may not enjoy violence in media, it has been established that some of the population has a predisposition to opt for and enjoy violence in media [12], particularly based on personality traits such as aggressiveness and risk-taking [13–14], everyday sadism [15], and arousal seeking tendencies [16]. Our studies especially concern those people who neither have a strong disposition for or against violence and can be swayed to opt for or against high violence, a group we refer to as the “middle group.” Specifically, we hypothesize that while people with certain traits and dispositions are more likely to opt for or against high violence, story contexts such as the fantasy genre, Nazi-historical story genre, and use of the third-person perspective can lead people without aggressive dispositions to opt for high violence.

2 How large is the group that can be influenced to choose violence under these different genre and perspective conditions?

We predict that a large portion of the population can be persuaded to opt for and enjoy violence under the conditions listed above.

3 How do different options for no, low, and high levels of violence affect the overall satisfaction with the story?

We reason that it is not predisposition alone that leads people to enjoy highly violent stories; it is also not control by itself (having a choice versus lacking a choice) that leads to higher satisfaction, even though both predisposition [17] and control [18] are factors that can lead to higher enjoyment. Instead, we suspect that *when* people opt for high violence in stories, they are more likely to enjoy the story. This hypothesis is especially relevant in context of Hypotheses 1 and 2. We conjecture that when those people from the middle group opt for high violence they will *as a result of their choice enjoy the story more than if they had opted for no or low violence*. By opting for violence, people opt for enjoyment.

4 Do people show signs of regret after violent choices?

In the context of our studies, we define regret as the wish to correct a previous aggressive choice by opting for an apology in future choices. Our study design of two consecutive choices of low and high violence is well suited to record patterns of regret as a first choice for high violence followed by an apology. Our hypothesis is that this choice pattern of regret is linked to low enjoyment. It is not well understood when the rules of morality are fully suspended in game playing and fiction and when they are merely overshadowed. We suggest that regret choice patterns indicate that moral judgment is not fully suspended, but rather only momentarily overshadowed in a previous choice. Regret also raises the question of whether the suspension of morality is a general effect of games and fiction or an individual choice of players and readers [19].

5 What is the relation of choice to satisfaction in violent interactive fiction?

We hypothesize that it is specifically the choice aspect of interactive fiction that drives satisfaction. Consequently, we hypothesize that when we make our narratives non-interactive, regardless of violence level satisfaction will be much lower than when participants are given the opportunity to make choices about how the story will precede. This would indicate that it is the choice for violence, not the violence alone, that accounts for enjoyment of violence in interactive narrative.

### The role of choice, control, and agency for satisfaction

A key factor for the enjoyment of violence is control and agency. In this study, we distinguish between control (having a choice) and agency (the feeling of control). Choice in media selection has been shown to influence reader reaction, with lack of choice increasing participant reactance to even preferred media when it is forced; having choices leads to higher satisfaction, though the effect is not always strong [18].

While our studies also concern the difference between choice and forced choice (equivalent to no choice), our focus is on the difference between specific choices for and against high violence. In a two-choice scenario between high and low violence, making a choice for high violence, rather than the choice against it, seems to indicate a higher degree of agency; put differently, the choice for high violence may feel more like agency. Our studies allow further insights how control and agency (feeling of control) interact.

Within media context, it has been shown that violent video games provide people, particularly adolescent boys, with a context to voluntarily control the emotional situations they confront, meaning that it is partially control (agency) that makes violent video games pleasurable [20–22]. In this model, feeling enjoyment from violence may rely on identifying violence as an indicator of situational moral disengagement that in turn allows for pleasurable identification with the violent character and a feeling of increased agency and accomplishment [23]. Similarly, Vaughan and Greenwood [24] hypothesize that people understand their engagement with fictional violence as a way to understand the real world, regulate arousal, and experience a just world.

### Morality, agency, and distance in enjoyment of mediated violence

Morality is a key inhibitor in enjoyment of violence, and Flesch [25] has suggested that most fiction is perceived as enjoyable when moral goals are accomplished and the bad guys get their comeuppance. Hartmann and Vorderer [26] show that within a violent video game, fighting for a just purpose, fitting violence into established order, and establishing the situation as “just a game” increase enjoyment and decrease guilt. However, there is a variety of strategies for moral disengagement, such as moral justification, euphemistic labeling, advantageous comparison, displacement or diffusion of responsibility, disregard or distortion of consequences, dehumanization, and attribution of blame [19, 27].

Aesthetic distance can overshadow morality, creating a path to moral disengagement that allows for enjoyment and pleasurable narrative control. Oatley [28] suggests that narrative fictions and games allow people to navigate through structures and settings distanced from reality, promoting emotion in the creative making of a world. Similarly, Koopman and Hakemulder [29] propose a “multi-factor model” of reading in which fictional narratives evoke aesthetic distance that allows for role-taking, empathy, and the suspension of judgement.

In the context of our study, factors that are likely to increase enjoyment of violence thereby include:

- breaking out of the ordinary world/taboo breaking;
- making a story more interesting or surprising;
- agency that comes from opting for violence [29];
- completion of narrative [19];
- accomplishment [23];
- eudaimonia [30];
- meaningfulness to the narrative [31–32];
- fictionality;
- eventfulness [33];
- specific genres and perspective (see our studies below).

However, it is not clear whether particular choices or the very fact that one has a choice drives enjoyment. We also suggest that regret, as a moral emotion that follows an earlier transgression, needs to be considered in the context of these factors (see Hypothesis 4). In general, desensitization over prolonged exposure to violence may increase individual enjoyment of violent media through moral disengagement regardless of initial traits [19, 34]. There seems to be a positive relationship between moral disengagement and playing violent video games through emotional desensitization [35–36].

Our studies cannot explain whether violent media contributes to aggressive behavior. Active participation in mediated violence as in game playing may produce different responses than passively viewing violent behavior. The General Affective Aggression Model [37] has been popularized to explain how playing violent videogames contributes to aggressive behavior.

### The choosing and enjoying of mediated violence

In this set of studies, we focus on interactive fiction in order to examine the question of choice. We offer our participants two consecutive choices in a short story between high, low, and no violence and we ask them questions concerning their satisfaction, involvement, and perception of morality. Our particular design of choices allows us to track how many people switch from one path to another. We suggest that people who switch from a highly violent first choice to an unviolent second choice (apology) show signs of regret. Specifically, we measure how many people make which choices and how they rate their satisfaction after making their choices under different genre and perspective conditions.

We created basic stories with variations in different genres and perspective conditions (second and third person). We reason that different genres create distinctive sets of expectations and levels of detachment that will have an influence on reader choice (Hypothesis 1), satisfaction (Hypothesis 3 and 5), likeliness for regret (Hypothesis 4), as well as the overall number of those for opt for violence (Hypothesis 2). We offer these stories with two consecutive plot choices (Study 1, 2, 3), no choice (Study 4), and with a single choice without showing outcomes of the choice to test anticipation (Study 5).

## Study 1

### Methods

**Fig 1:**
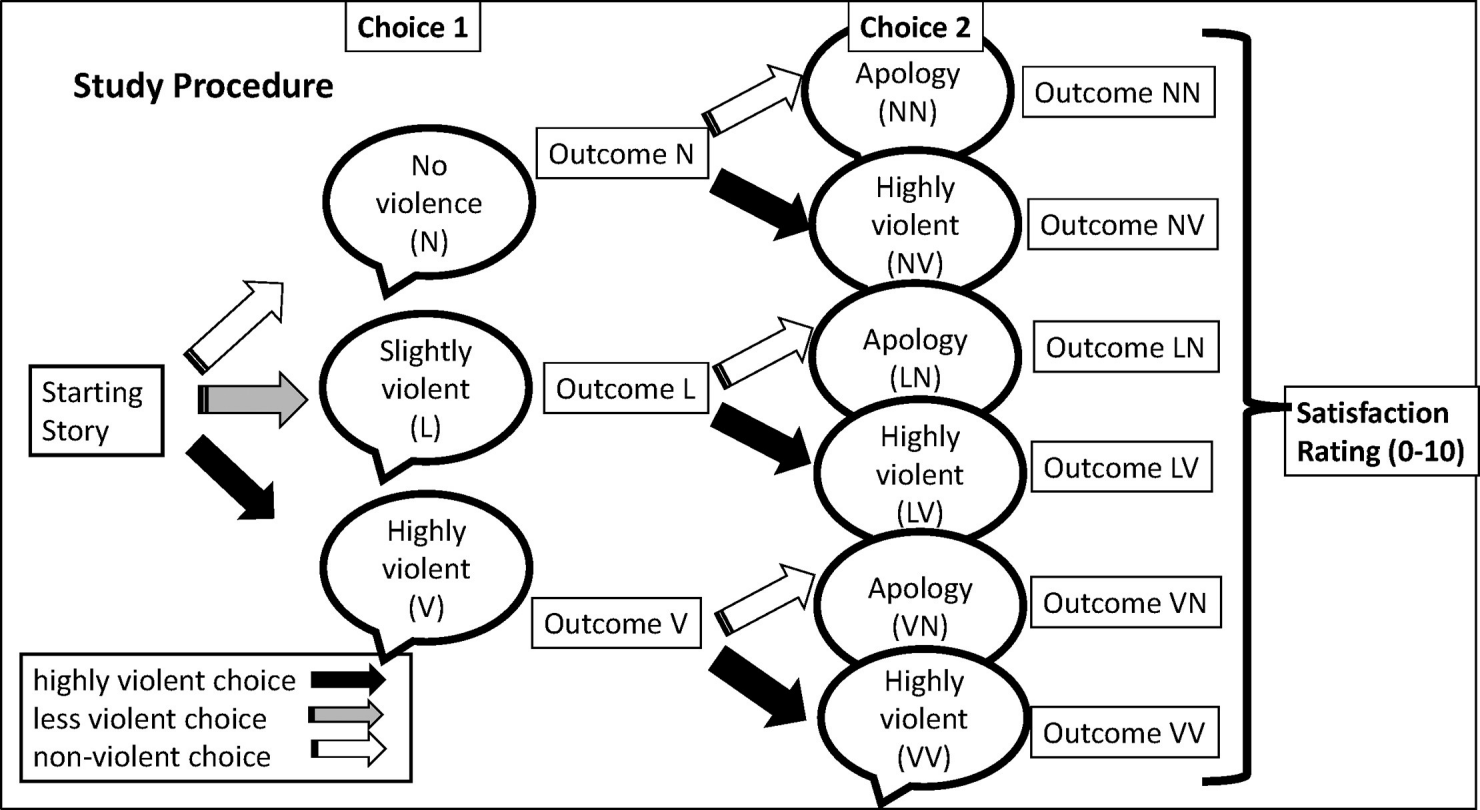
Presented is the order of the tasks for the participants, beginning with making choice 1, choice 2 to providing a satisfaction rating.

In this study, participants received a short story and were told to “read the following story” and then twice during the story were asked to “choose what comes next.” Each story introduced two main characters in conflict with one another. One of these characters is given a motive for being annoyed with the other character, such as a noise disturbance. After 3–4 sentences describing an encounter between these characters, participants were given their first choice of three options about how the story would continue. One choice was not violent, usually starting a conversation about topics that were not related to the subject of disturbance, the second involves a low level of violence, such as a slap, and the last one was highly violent, such as hitting the other character with a baseball bat. After making the first choice, participants were directed to a sentence depicting the consequences of the action, describing either the follow-up of the mundane conversation, or the confusion of the other character from the low violence action, or the injury of the other character from high violence. After that, participants made a second choice for the aggressive character between a highly violent or non-violent action. The non-violent choice could be ending the conversation peacefully (if following a first non-violent choice) or an apology if it followed a prior act of violence. This means that there are six patterns of choices by participants resulting from no violence (= N) low violence (= L) and high violence (= V): first no violent choice and end of the conversation (= NN); first no violent choice and then performance of a violent action (= NV); first slightly violent and then apology (LN); first slightly violent and then escalation (LV); first highly violent and then apology (VN); and first highly violent and then escalation (VV), see Fig 1.

After the story, the participants were asked “how satisfied do you feel with the events of the story?” They were given a scale from 0 to 10 with 0 being not satisfied and 10 very satisfied. We collected only whole numbers. For each story we created four versions that each fell into one of the following genres: realistic-contemporary, fantasy, Nazi-historical, and general-historical. We selected these genres since we reasoned that they would direct participants to either more positive (fantasy), expectation-congruent (Nazi-historical), negative (general-historical), or expectation-incongruent and negative (realistic-contemporary) attitudes about violence. In each version, we preserved the basic plot with the reason for the original annoyance (i.e. noise disturbance); the relationship between the characters, such as being neighbors; and the choices of violent actions, such as stabbing. The violent actions were identical or highly similar, (i.e. stabbing with a pocket knife versus a dagger) and the different genres featured appropriate settings. For each version, we also created second and third-person variations. All in all, we created three different stories (with different motivations and forms of violence), each having four different genre versions and two different perspective variations, resulting in a total of 24 different texts (see S1 Interactive Stories for all story versions).

We recruited participants via Amazon Mechanical Turk. For each of the 24 conditions, we recruited 21 to 23 participants, resulting in a total of 507 participants. Average age was 38 years; we had 255 female and 249 male participants with the rest preferring not to state their gender. We paid all participants at an approximate rate of $6/hour.

#### Statistics: Kruskal–Wallis one-way ANOVA and multinomial logistic regression

To analyze the variance of participants’ satisfaction across story path outcomes throughout the paper we employed a Kruskal–Wallis one-way analysis of variance, followed by a Dunn post-hoc test with the Bonferroni method for p-value adjustment. We turned to this non-parametric test because our data are ordinal and not normally distributed (many satisfaction ratings clumped at the ends of the scale, 0 and 10), violating the assumptions of the standard one-way parametric ANOVA.

Additionally, we were interested in the potential influence of story genre and story perspective on participants’ story path outcomes, which is a classification problem with two nominal predictors. To answer these questions, we used multinomial logistic regression, which can be thought of as an extension of logistic regression featuring a nominal dependent variable with more than two levels. NN, fantasy, and second-person were used as the reference categories for story path, genre and perspective. The analyses described here were reused in Study 2 and 3. All analyses were performed in R: the multinomial regression made use of the mlogit package, and the Dunn post-hoc test used the PMCMRplus package, while the rest of the analyses were carried out with R’s base package.

### Results

#### Effect of story path on satisfaction

Out of 507 total participants, 283 (56%) made a non-violent choice then continued with no violence (NN), 56 (11%) made two high-violence choices (VV), while 168 (33%) made at least one violent option (L or V), but did not opt twice for high violence, see Table 2, S1 Table. Overall, participants reported the highest average satisfaction in the VV story path (7.09), see Table 3. Participants who opted twice for no violence showed an average satisfaction of 6.71. 131 participants switched from a violence first choice (V or L) to a second choice of no violence (VN, LN) and showed an average satisfaction of 5.82. In comparison, only 37 participants increased violence after a first choice of N or L to a second choice of high violence (NV, LV). However, if they did switch in this way, their satisfaction was higher at an average of 6.65.

**Table 2:** Choice path distribution by genre and perspective (N = 507).

		Hist. Gn	Realist	Hist. Nazi	Fantasy	2^nd^ Person	3^rd^ Person	All
Participants	NN	81	92	65	45	154	129	283
	LN	24	16	38	16	47	46	93
	VN	4	5	7	22	14	24	38
	NV	2	3	3	3	5	6	11
	LV	10	4	6	6	12	14	26
	VV	7	7	10	32	19	37	56

*Note*. Shown are numbers of participants who made choices (N, L or V) within given story conditions.

**Table 3:** Satisfaction by choice path and genre (N = 507).

		Hist. Gn	Realist	Hist. Naz	Fantasy	2^nd^ Person	3^rd^ Person	All
Satisfaction	NN	6.81	6.74	6.52	6.76	6.44	7.05	6.71
	LN	6.22	6.44	5.24	5.63	6.11	5.39	5.75
	VN	8	7.2	5.71	5.45	6.07	5.96	6
	NV	5	8.33	3.66	6.33	7.2	4.83	5.91
	LV	6.7	7.75	6.33	7.5	6.08	7.71	6.96
	VV	7.29	6.57	6.6	7.31	8.42	6.41	7.09

*Note*. Shown are satisfaction rate by participants who made specific choices (N, L or V) within given story conditions.

A Kruskal–Wallis test yielded a significant effect of story path on participant satisfaction, χ2(5) = 17.74, p < .01. A Dunn post-hoc test using the Bonferroni correction for p-values revealed two significant contrasts. Participants in the VV story path (*M* = 7.09, *SD* = 2.9) were significantly more satisfied than participants in the LN story path (*M* = 5.75, *SD* = 2.65), *p* < .01. And participants in the NN (*M* = 6.71, *SD* = 2.46) story path were also significantly more satisfied than participants in the LN story path, *p* < .05. The tests revealed no other significant differences in satisfaction between the conditions.

#### Effect of genre and perspective on story path

We performed a multinomial logistic regression to determine whether story genre and perspective influenced participants’ choice of story paths. Table 4 presents the full results of the regression, but there are several significant effects of note. The general-historical, Nazi-historical, and realistic genres each yielded significantly less VV and VN story outcomes relative to NN (Nazi historical-VN, p < .01; all other effects, p < .001). 32 of 125 participants (26%) in fantasy opted for VV, compared to just 24 of 383 (6%) in the other genre conditions.

**Table 4:** Results of multinomial logistic regression: effect of genre and perspective on story path (six outcomes).

				95% CI for odds ratio		
		*B* (*SE*)	Odds Ratio	Lower Bound	Upper Bound	p-value
LV vs NN	Intercept	-1.13 (.30)	0.32	0.17	0.59	< .001***
	GenreGenHist	-.21 (.38)	0.80	0.39	1.68	.562
	GenreNazi	0.51 (0.36)	1.66	0.83	3.33	.155
	GenreRealistic	-0.71 (0.40)	0.49	0.23	1.07	.075
	GenreFantasy		Reference			
	Perspective3rd	0.17 (0.24)	1.19	0.74	1.92	.479
	Perspective2nd		Reference			
LV vs NN	Intercept	-2.19 (0.47)	0.11	0.04	0.28	< .001***
	GenreGenHist	-0.09 (0.55)	0.92	0.31	2.70	.875
	GenreNazi	-0.38 (0.61)	0.69	0.21	2.27	.539
	GenreRealistic	-1.1 (0.67)	0.32	0.08	1.20	.092
	GenreFantasy		Reference			
	Perspective3rd	0.37 (0.41)	1.44	0.64	3.24	.377
	Perspective2nd		Reference			
NV vs NN	Intercept	-2.19 (0.67)	0.05	0.01	0.20	< .001***
	GenreGenHist	1.01 (0.93)	0.37	0.06	2.28	.281
	GenreNazi	-0.38 (0.84)	0.68	0.13	3.56	.653
	GenreRealistic	-0.73 (0.84)	0.48	0.09	2.49	.384
	GenreFantasy		Reference			
	Perspective3rd	0.41 (0.62)	1.50	0.44	5.07	.511
	Perspective2nd		Reference			
VN vs NN	Intercept	-1.17 (0.33)	0.31	0.16	0.59	< .001***
	GenreGenHist	-2.34 (0.58)	0.10	0.03	0.30	< .001***
	GenreNazi	-1.56 (0.48)	0.21	0.08	0.54	.001**
	GenreRealistic	-2.25 (0.53)	0.11	0.04	0.30	< .001***
	GenreFantasy		Reference			
	Perspective3rd	0.87 (0.37)	2.40	1.15	4.98	.019*
	Perspective2nd		Reference			
VV vs NN	Intercept	-0.87 (0.30)	0.42	0.24	0.75	.003**
	GenreGenHist	-2.17 (0.46)	0.11	0.05	0.28	< .001***
	GenreNazi	-1.59 (0.42)	0.20	0.09	0.46	< .001***
	GenreRealistic	-2.30 (0.46)	0.10	0.04	0.25	< .001***
	GenreFantasy		Reference			
	Perspective3rd	1.00	2.72	1.43	5.13	.002**
	Perspective2nd		Reference			

*p< .05

**p< .01

***p< .001.

McFadden R^2 = .07.

We observed a similar effect of perspective, where relative to the second-person perspective, the third-person perspective yielded more VV and VN story outcomes relative to NN (for VV, p < .01; for VN, (p < .05). 37 of 256 participants (14%) opted for VV in third-person compared to 19 of 252 in second-person (8%); and 24 of 256 participants (9%) opted for VN in third-person compared to 14 of 252 in second-person (6%).

In other words, the fantasy genre influenced participants to choose VV and VN more frequently and NN less frequently when compared with all other genres. And the third-person perspective likewise influenced participants to choose VV and VN more frequently and NN less frequently than the second-person perspective.

#### Middle group

Given the choice patterns in this study, we can distinguish three populations of participants:

1. People with a strong inclination for morality and/or against violence in all story settings that our conditions cannot persuade to opt against their disposition for the least violent pattern offered (NN in Study 1).
2. A middle group who might opt for a choice of high violence in some story condition, but for no violence in others.
3. People with a strong inclination for violence that our conditions cannot persuade to opt against their disposition and preference and who will opt for the most violent path (VV) (for people with predisposition to appreciate violence, see [15, 16, 38]).

Our data allows us to estimate the size of this middle group. We assume that the participants who opt for either extreme (NN and VV) most represent the portion of the population that is unaffected by story genre and perspective. By finding the smallest proportion of participants who follow these extreme paths, we can estimate the size of the population that has strong predispositions for or against violence. By subtracting this percentage from 100, we can then estimate the size of the population that can be influenced by story condition. The conditions with the fewest people opting consistently for high violence (VV) are second-person general-historical and second-person Nazi-historical, both of which had 1/63 people opting for the VV path (2%). The condition with fewest people making a non-violence choice (NN) is in third-person fantasy, with 20/62 opting for the NN path (32%). Consequently, we can estimate that 66% of our participants can be influenced by genre and perspective.

#### Gender

There was no clear difference between males and females in choice and satisfaction. 146 females opted for NN and expressed a satisfaction of 6.86 compared to 135 males who rated their satisfaction as 6.67 in average. 28 females and 28 males chose VV, the females rated their satisfaction as 7.04 and the males as 7.14 in average. (Note again that 3 people preferred not to state gender).

### Discussion

The astonishing finding of Study 1 is the link between high violence and high satisfaction. Overall, the study has five main findings:

1. People were statistically significantly more likely to make highly violent choices in the fantasy genre than in any of the other genres.
2. People were also significantly more likely to opt for VV in third-person perspective stories and more likely to opt for NN in second-person perspective.
3. Participants who made only violent choices expressed notably higher levels of satisfaction with the story.
4. Participants were more likely to show regret by moving from violence to no violence than to move from lower to higher violence. If they switched to no violence they showed notably lower satisfaction.
5. There was a large middle group of 66% of the participants who could be persuaded to opt for violence.

The unequal distribution of choice paths by genre and perspective indicates that it is not predisposition or specific traits alone that influence choice and satisfaction. Within the study design, it is likely that participants without a strong disposition for or against high violence reacted to both inhibitors and enablers for making violent choices. Second-person conditions, realistic and general-historical settings had an inhibiting effect, perhaps a reminder of personal responsibility and morality. Contrastingly, third-person and fantasy conditions, but not Nazi-historical stories, had an enabling effect, emphasizing fictionality and consequently allowing taboo breaking. Even though violence is expectation congruent for the Nazi-historical genre, unlike the fantasy genre, the Nazi-historical genre did not increase the number of highly violent choices made. Perhaps participants wanted to take the opportunity to have a Nazi character behave more morally than what would be expected in a story; or they may have wanted to distance themselves from the Nazi characters, and thus make choices that align with their own moral behavior. Note again that the stories were quite graphic in the descriptions of violence, including in fantasy, with conclusions such as you “take a piece of glass from the broken jar and cut his throat,” see S1 Interactive Stories.

Given that highly violent choice paths are linked to higher satisfaction, there is an intriguing possibility that choice for violence is a choice for satisfaction. Our next studies aim to clarify this possibility. In our next studies, we aim to investigate under what conditions and to what extent this middle group can be persuaded to choose highly violent choices in narrative. If we can indeed persuade more people to opt for high violence, we want to know whether these people display the same high satisfaction after making the choice.

## Study 2

We wanted to know if more people opt for high violence when the option against violence is not available and the choice is only between low and high violence. We also wanted to know how people who can be persuaded to opt for high violence in certain conditions, such as limited choice, rate their enjoyment. Do these people still rate high violence as highly satisfying or do they show low satisfaction?

### Methods

We repeated Study 1 without the no-violence option in the first choice, using the same basic 24 stories (see S1 Interactive Stories). For each of the 24 conditions, we recruited 19 to 21 participants, resulting in a total of 480 participants. The average age of participants was 34. 251 reported as female and 226 as male, with the remaining preferring not to state gender.

**Fig 2:**
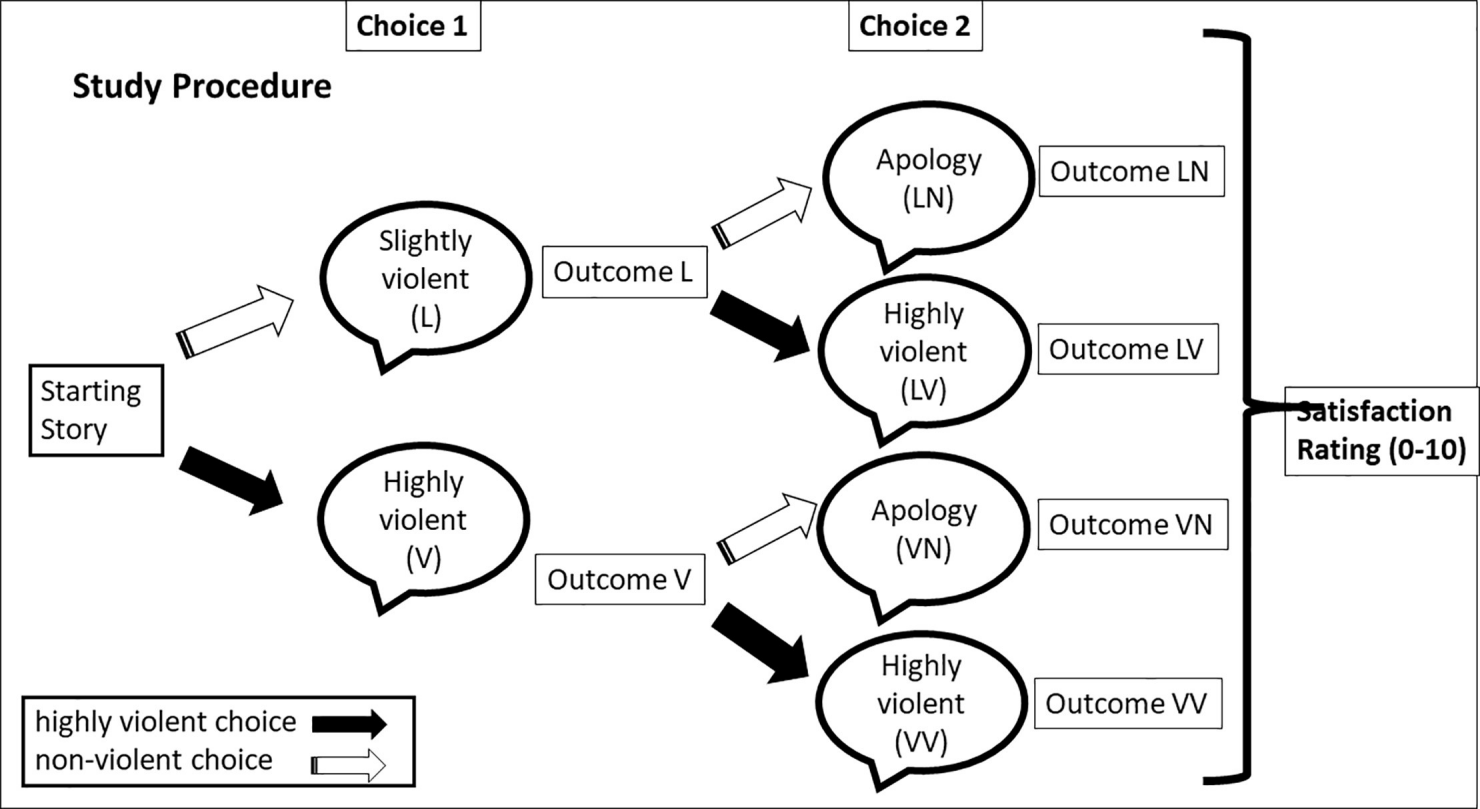
Presented is the order of the tasks for the participants for Study 2, beginning with making choice 1, choice 2 to providing a satisfaction rating.

### Results

#### Effect of story path on satisfaction

Out of 480 total participants, 315 (66%) made a low-violence choice then apologized (LN), and 78 (16%) made two high-violence choices (VV). 65 (14%) switched from a high violence choice to a second choice of no violence (VN). In comparison, only 22 (5%) increased violence after a first choice of low violence to high violence (LV), see Table 5, S2 Table.

**Table 5:** Choice path distribution by genre and perspective (N = 480).

		Hist. Gn	Realist	Hist. Naz	Fantasy	2^nd^ Person	3^rd^ Person	All
Participants	LN	89	83	81	62	168	147	315
	VN	12	17	14	22	34	31	65
	LV	6	6	6	4	9	13	22
	VV	10	18	19	31	32	46	78

*Note*. Shown are numbers of participants who made choices (L or V) within given story conditions.

Overall, participants reported the highest average satisfaction in the VV story path (6.5). The lowest averages occurred in second choice low violence (4.48 LN, 4.32 VN), see Table 6.

**Table 6:** Satisfaction by genre and perspective (N = 480).

		Hist. Gn	Realist	Hist. Naz	Fantasy	2^nd^ Person	3^rd^ Person	All
Satisfaction	LN	4.44	4.28	4.68	4.53	4.24	4.74	4.48
	VN	2.83	5.7	3.93	4.32	3.94	4.74	4.32
	LV	6.33	4.17	6.5	7.5	6.67	5.54	6.0
	VV	6.6	7.18	6.32	6.26	6.84	6.3	6.53

*Note*. Shown are average satisfaction ratings on a scale from 0 to 10.

A Kruskal-Wallis test yielded a highly significant effect of story path on participant satisfaction, χ2(3) = 35.77, p < .001. A Dunn post-hoc test using the Bonferroni correction for p-values revealed two highly significant contrasts. First, between participants who chose VV (*M* = 6.53, *SD* = 2.75) and LN (*M* = 4.48, *SD* = 2.73), p < .001. And second, VV and VN (*M* = 4.32, *SD* = 2.9), p < .001.

#### Effect of genre and perspective on story path

As in Study 1, we performed a multinomial logistic regression to determine whether story genre and perspective influenced participants’ choice of story paths. LN, fantasy, and second-person were used as the reference categories for story path, genre and perspective. Table 7 presents the full results of the regression, but there are several significant effects of note. The general-historical, Nazi-historical, and realistic genres each yielded significantly less VV story outcomes relative to LN (Nazi historical and realistic, p < .05; general-historical, p < .001). 31 of 119 participants (26%) in fantasy opted for VV, compared to just 47 of 361 (13%) in the other genre conditions. A final significant effect of genre was the lesser likelihood of LV appearing in general-historical stories than fantasy. 12 of 117 participants (10%) in general-historical opted for LV, compared to 22 of 119 (18%) in fantasy.

**Table 7:** Results of multinomial logistic regression: Effect of genre and perspective on story path (four outcomes).

				95% CI for odds ratio		
		*B* (*SE*)	Odds Ratio	Lower Bound	Upper Bound	p-value
LV vs LN	Intercept	-1.06 (.28)	0.34	0.20	0.60	< .001***
	GenreGenHist	-0.97 (0.39)	0.38	0.17	0.82	.014*
	GenreNazi	-0.7 (0.38)	0.49	0.23	1.03	.059
	GenreRealistic	-0.55 (0.36)	0.58	0.28	1.18	.131
	GenreFantasy		Reference			
	Perspective3rd	0.05	1.06	0.61	1.80	.843
	Perspective2nd	(0.27)	Reference			
VL vs LN	Intercept	-3.00 (0.57)	0.05	0.02	0.15	< .001***
	GenreGenHist	0.02 (0.67)	1.03	0.28	3.80	.965
	GenreNazi	0.13 (0.67)	1.14	0.23	1.03	.840
	GenreRealistic	-0.11 (0.67)	1.12	0.28	1.18	.866
	GenreFantasy		Reference			
	Perspective3rd	0.5 (0.45)	1.65	0.61	1.81	.263
	Perspective2nd		Reference			
VV vs LN	Intercept	-0.96 (0.26)	0.38	0.23	0.64	< .001***
	GenreGenHist	-1.5 (0.40)	0.22	0.10	0.49	< .001***
	GenreNazi	-0.76 (0.33)	0.47	0.24	0.91	.025*
	GenreRealistic	-0.84 (0.34)	0.43	0.22	0.85	.015*
	GenreFantasy		Reference			
	Perspective3rd	0.52 (0.26)	1.68	1.00	2.79	.048*
	Perspective2nd		Reference			

*p< .05

***p< .001.

McFadden R^2 = .03.

Study 2’s multinomial regression also found a similar effect of perspective as Study 1. Relative to the second-person perspective, the third-person perspective yielded more VV story outcomes relative to LL, p < .05. 46 of 237 participants (19%) opted for VV in third-person compared to 32 of 243 in second-person (13%); and 13 of 237 participants (5%) opted for VN in third-person compared to 9 of 243 in second-person (4%).

In summary, a general similarity was established with Study 1’s findings. The fantasy genre and third-person perspective influenced participants to choose VV more frequently and LN less frequently.

#### Middle group

We calculated the middle group as in Study 1. The middle group was 47%.

#### Gender

Females were less likely to opt for high violence than males and on average expressed lower satisfaction in all conditions. 181 women (satisfaction: 4.13) and 135 males (satisfaction: 4.95) opted for LL; while only 22 women (satisfaction: 5.35) but 55 men (satisfaction: 6.96) opted for VV. More women (39) switched from high violence to apology (VL) than men (26).

### Discussion

The main finding of Study 2 is the fact that we can indeed make the middle group shift towards a choice for high violence. Overall, the study confirms four of study 1’s main findings:

1. People were again statistically significantly more likely to make highly violent choices in the fantasy genre than in any of the other genres.
2. People were also again significantly more likely to opt for VV in third-person perspective stories and more likely to opt for LN in second-person perspective.
3. Participants who made only violent choices again expressed notably higher levels of satisfaction with the story, especially if their last option was for high violence.
4. Few people changed their choice path, in line with theories of cognitive dissonance [39]. Those who did showed regret (switch to lower violence, paired with low satisfaction).

The large middle group seems to have been persuaded to opt for high violence. The size of the middle group within the conditions of Study 2 was again high at 47%, but lower than in Study 1 that offered more choice options. Specifically, whereas in Study 1 11% (56 of 507) of participants chose the VV option, 16% (78 of 480) chose this option in Study 2. Consequently, by eliminating the non-violent option in the first choice we were able to persuade more participants to choose high violent options in Study 2. People seem to be swayed not only by narrative conditions but by how their choice is framed as well. To emphasize again, the satisfaction ratings for this VV group remain generally consistent across studies despite these differences in choice path distribution. However, unlike in Study 1, the participants in Study 2 who made more violent choices expressed significantly higher levels of satisfaction with the story than any other group, especially if their last option was for high violence. A likely explanation for this increased difference is that since we offered participants a forced choice with no non-violent option in Study 2, it is likely that many participants felt dissatisfied. These differences in choice path distribution along with the consistencies in satisfaction ratings seem to indicate that it is the choice for violence and not predisposition alone that drives satisfaction.

There is a difference in gender between Study 1 and Study 2. While Study 1 records no clear differences, females in Study 2 were notably less satisfied. This might suggest that females had a stronger preference and reacted strongly to more limited choices and forced choices for some violence (L or V), while males were more flexible to adjust without being dissatisfied.

## Study 3

### Methods

We created a follow-up study with no participant choice for plot development. Participants were randomly given a completed version of the story with the choices already made. We created 32 versions of one of the stories, namely all four paths for the realistic, fantasy, Nazi-centered historical, and general-historical genre with either second- or third person-perspective, as in Study 2, but wrote each of the possible outcomes as part of that story. For example, a participant would receive a second-person, realistic story with the slightly violent action followed by an apology (LN) already woven into the story. Participants read one of these narratives and immediately afterwards rated their satisfaction with the story on a scale from 0 to 10. Participants were randomly assigned a story version. Each of the 32 story conditions (4 story paths x 4 genre x 2 perspectives) was rated by 8–11 participants, for a total of 295 different raters. All three base stories behaved similarly in Study 2, and we selected the story that correlated most closely to the overall pattern of satisfaction in the genre categories and perspective conditions (Pearson correlation of r = .76, n = 480) compared to the average satisfaction for all paths).

#### Statistics: Ordinal logistic regression

We combined the data from Study 2 (choice) and Study 3 (no choice) for our analyses of the influence of participant choice on satisfaction. We chose an ordinal-logistic-regression model where the nominal variables of choice presence, participants’ story choices, and their interaction were used to predict ordinal satisfaction ratings. The choice being present was used as the reference for the choice variable. LN was used as the reference category for story choice. The ordinal logistic regression was performed in R with the MASS package.

### Results

#### Effect of reader choice on satisfaction

Participants who had no choice in the story progression consistently reported lower average satisfaction with the highly violent stories VV (*M* = 2.27, *SD* = 2.58) than with the less violent stories LN (*M* = 3.87, *SD* = 2.53), see Table 8, S3 Table. An ordinal logistic regression found several significant main effects of choice and story path but these were overshadowed by significant interaction effects. When choice was absent, participants satisfaction dropped for the VN, LV, and VV stories (p < .05, p < .001, p < .001). In short, satisfaction dropped for each of the non-LN stories when choice was absent.

**Table 8:** Results of ordinal logistic regression: Effect of reader choice and story path on satisfaction.

			95% CI for Odds Ratio		
	*B* (*SE*)	Odds Ratio	Lower Bound	Upper Bound	p-value
No Choice	-0.83 (0.25)	0.44	0.27	0.71	< .001***
LV	0.85 (0.49)	2.34	0.91	6.16	.080
VN	0.27 (0.31)	1.31	0.72	2.39	.373
VV	1.03 (0.30)	2.81	1.55	5.12	< .001***
No Choice * LV	-1.97 (0.57)	0.14	0.05	0.42	< .001***
No Choice * LV	-0.93 (0.42)	0.39	0.17	0.89	.026*
No Choice * VV	-2.29 (0.42)	0.10	0.04	0.232	< .001***

*p< .05

***p< .001.

**Fig 3:**
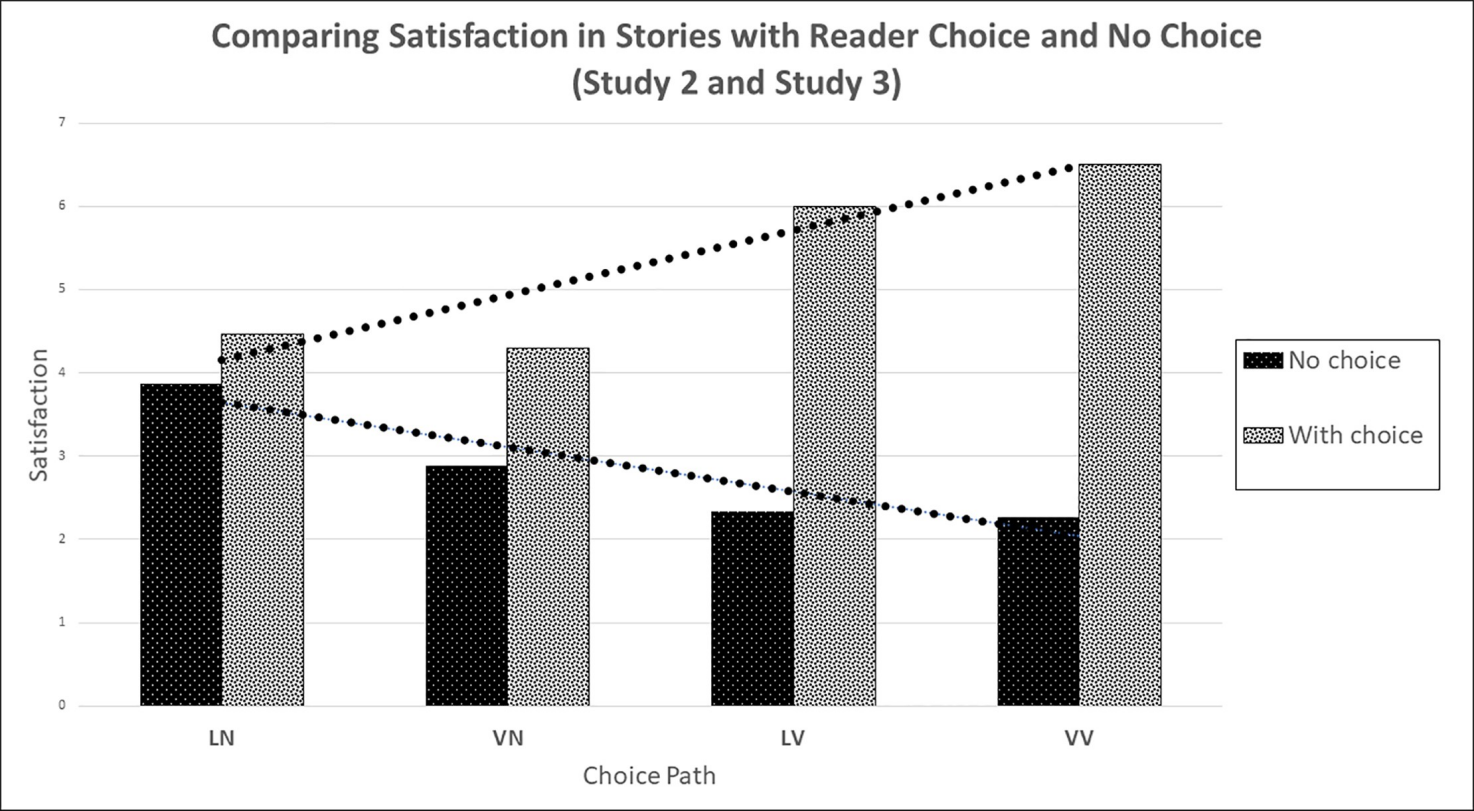
Satisfaction ratings by participants who had choices or had no choice in the plot development of the story, presented by story path (low and high violence). Shown are the data from Studies 2 and 3.

This reverses the pattern we found in Study 2 where participants who had a choice in story paths expressed higher satisfaction with high violence, see Fig 3. While participants in Study 2 reported an average satisfaction of 6.53 after making highly violent choices, participants in Study 3 who read the same highly violent stories but without a say in the outcome of the story expressed a much lower average satisfaction of 2.27. In general, participants who had choices reported being much more satisfied. There was a small subgroup of 14 participants for the high-violence condition (VV) that showed a satisfaction rating above 5 (N = 74), while 50 participants gave ratings of 0–2.

#### Gender

In Study 3 with no choices, females showed lower satisfaction in any group. For LN, satisfaction was 3.14 for females and 4.3 for males. In VV it was 1.78 for females and 2.88 for males.

### Discussion

Study 3 shows a strong pattern: When there is no choice and participants read completed stories, they express very low satisfaction with highly violent stories. This is in contrast to Study 2 where participants expressed higher satisfaction after making highly violent choices, even though the resulting stories were identical to the ones in Study 3, see Table 8.

What should one make of this stark contrast? Choice-making has a central influence on satisfaction. This is in line with previous studies that have found that control of the narrative seems particularly linked to enjoyment of violence [20, 21, 23]. Furthermore, the high satisfaction ratings that follow highly violent choices could reflect an appreciation of one’s own impact on the story [20, 21, 22]. It is also possible that by making a choice for high violence, people opt for the non-realistic choice to create aesthetic distance. Reversely, those in Study 1 and 2 who initially opted for low violence likely felt that this choice was not actually a choice for low violence, but rather a choice against violence, and thus felt similar to the conditions of this study that offered no choice. Consequently, their low satisfaction ratings were based both on their low appreciation of the story and their dissatisfaction with the inability to shape the story. To further clarify these options, we created Study 4.

As indicated, a minority of participants (14 of 74 or 19%) still expressed higher satisfaction with the high violence condition in Study 3, representing a group that enjoys violence regardless of condition or presence of agency.

Females showed notably lower satisfaction in Study 3 with no choices in all story paths, while there was no clear difference in gender in Study 1. Choice seems thus to play a larger role for satisfaction for females than males.

## Study 4

We wanted to know at which point participants decide on their ratings of satisfaction—before or after seeing the outcomes of their choices—and how they explain their choice making.

### Methods

We created a survey based on Study 2 with one addition: right after making their initial choice and before seeing the outcome of that first choice, we inserted a question concerning anticipated satisfaction and several questions about the reasons why participants made their choice between the high or low violence option. We could not include these questions in Study 1 or 2 since such a question would have likely resulted in priming effects for the later tasks. We first asked participants to “predict how satisfied you will be with the story based on your choice for how the story should continue.” Afterwards, participants were asked: “To which degree did each factor influence your choice about how the story should continue?” They ranked the following on a sliding scale from 0 to 10 as in the other studies with the quoted explanations: realism (“the story would be realistic”), fun (“the story would be fun”), morality (“the story would be less immoral”), curiosity (“I am curious to see what comes next”), violence (“I am interested in violence”), and distaste (“I dislike blood and brutality”). As with Study 3, we used the eight versions of Story 2 because it correlated most closely to the overall pattern of satisfaction in the genre categories and perspective conditions. 241 participants rated one of the eight story conditions, with 33–57 for each genre condition.

#### Statistics: Wilcoxon rank-sum test and logistic regression

To analyze the influence of participants’ first story choice (L or V) on satisfaction we employed a Wilcoxon rank-sum test. As with the non-parametric tests used in Studies 1 and 2, the Wilcoxon test best fit our ordinal and non-normally distributed data.

Additionally, we wanted to determine whether participants’ ratings of their motivations could predict whether they made a L or V first choice. And this question is well suited for a logistic regression model where the binary first choice is predicted by the ordinal motivation ratings (treated as continuous). All analyses were performed with R’s base package.

### Results

#### Effect of first story choice on predicted satisfaction

Participants were asked to rate their satisfaction with the story after making their first choice and before seeing the outcome of their choice. 63 participants chose the highly violent first option and reported a higher level of anticipated satisfaction (*M* = 5.97, *SD* = 2.68) than the 178 participants who chose the less violent option (*M* = 4.29, *SD* = 2.92) (Table 9, S4 Table).

**Table 9:** Choice influence for L and V, genre, and perspective (N = 241).

	Predict. Satisfac.	Fun	Curiosity	Realism	Morality	Interest in Vio.	Distaste for Vio.
L	4.29	3.66	5.2	4.98	5.5	2.55	5.54
V	5.97	5.52	6.11	4.95	3.08	3.62	3.05
His.Gen	4.05	3.38	5.21	5.05	4.81	2.8	5.5
Realistic	4.2	3.78	4.87	4.5	4.37	2.93	5.17
His.Nazi	4.8	3.3	5.2	4.85	5.28	2.3	4.9
Fantasy	5.85	6.15	6.5	4.68	3.78	3.3	4
2^nd^ Per.	4.72	4.17	5.3	4.82	4.96	2.8	5.13
3^rd^ Per.	4.74	4.13	5.58	5.12	4.3	2.86	4.66

*Note*. Participants first rated their predicted satisfaction with the story after making their first choice, but without seeing any outcomes. Then they rated what influenced their choice.

A Wilcoxon rank-sum test revealed that this difference was significant, *W* = 2429, *p <* .001. Consequently, we have strong evidence that overall satisfaction was a product of anticipation and largely determined prior to seeing the outcome of the story choices.

In addition, we asked participants to rate potential motivations for making the choice that they did, and we inputted this data into a logistic regression as continuous measures to predict their binary first choice, see Table 10. The results of the regression show that higher *fun* ratings reliably predicted the highly violent choice (p < .01), while higher *distaste for blood and brutality* ratings reliably predicted the less violent choice (p < .001). However, *realism*, *moral motivation*, *curiosity*, *liking violence* did not reliably predict story choice. Of particular note: the fact that moral motivation did not predict story choice suggests that participants did not see the story choice as a moral situation; and that *liking violence* was not a reliable predictor indicates that participants who chose the highly violent option do not possess or were not motivated by an inherent preference for violence.

**Table 10:** Results of logistic regression: Predicting violent choices with self-reported motivations.

			95% CI for odds ratio		
	*B* (*SE*)	Odds Ratio	Lower Bound	Upper Bound	p-value
Intercept	-0.58 (0.56)	0.56	0.18	1.65	.300
Realism	0.06 (0.06)	1.06	0.94	1.20	.365
Fun	0.20 (0.07)	1.21	1.07	1.40	.004**
Morality	-0.11 (0.7)	0.90	0.78	1.03	.116
Curiosity	-0.04 (0.08)	0.96	0.83	1.11	.630
Like Violence	0.10 (0.07)	1.10	0.96	1.27	.166
Distaste	-0.30 (0.08)	0.73	0.63	0.85	< .001***

**p< .01

***p< .001.

Hosmer and Lemeshow R^2 = .22

Cox and Snell R^2 = .23

Nagelkerke R^2 = .33.

#### Gender

Females anticipated notably lower satisfaction than males. In L, 76 females predicted a satisfaction of 3.27 and 99 males 5.12 in average. In VV, 26 females predicted a satisfaction of 5.08 and 37 males 6.59.

### Discussion

The ratings show that as soon as they make their first choice people predict a significantly higher satisfaction. This indicates that satisfaction does not arise solely from seeing consequences of their decisions. Consequently, it suggests that the satisfaction cannot alone be explained by prediction-reward theory since participants did not see the outcomes.

People who opted for the low violence option reported being guided by morality and distaste for violence and anticipated low satisfaction. People who opted for high violence reported being guided by fun and not morality and anticipated high satisfaction.

The ratings for realism are close to equal in both conditions. Hence, there is no evidence of a detachment from reality in making the decision for high violence or difference in satisfaction. In this respect our finding differs from suggestions of Waddell [30] who showed that induced senses of realism lead to lower enjoyment. In contrast to our study, Waddell [30] did not offer participants choices.

That means the first decision is already a decision about having fun and to discount morality, or vice-versa. This is important to emphasize since it provides a motivation for people who choose high violence. People who choose high violence *decide* to have fun, *decide* to discount morality, and make it happen in their decision. Those who choose the most violent option indicate that morality is not as high of a concern, supporting the disengagement theory [34, 39, 36].

## Overall discussion

People who chose highly violent plot developments generally enjoyed the stories more than people who made less violent choices. A large percentage of our participants (up to 66%) could be persuaded to opt for highly violent plot choices and as a result rated their satisfaction higher than people who did not make highly violent choices. However, people who did not have choices and read completed stories generally strongly disliked high violence. It is when participants are given control of a situation and execute it by opting for high violence that they find greater satisfaction. In short, choosing violence increases enjoyment.

Specifically, our studies have five findings:

1. What are the conditions under which people are more likely to opt for highly violent plot developments? *Fantasy and third-person perspectives enabled people to choose high violence*, *while realistic and historical genres and second-person perspectives acted as deterrents of highly violent choices*. Hypothesis 1 was partly supported. We did not, however, predict that the Nazi-historical genre would not enable highly violent choices.
2. How large is the group that can be influenced to choose violence under these different genre and perspective conditions? *In our studies*, *the middle group was the largest of all groups*. *Depending on the study conditions*, *up to 66% of participants could be influenced to make different choices according to story condition (Study 1)*.
3. How do different options for no, low and high levels of violence affect the overall satisfaction with the story? *Generally*, *people who opt for highly violent plot choices were more satisfied than people who opt for low violent options (Studies 1*, *2*, *4)*. Moreover, satisfaction for the VV group remains high even when more people opt for high violence (VV) under different story or choice conditions. Hypothesis 3 was supported.
4. Do people show signs of regret after violent choices? *Regret plays a substantial role in decreasing satisfaction*. It is much more common for people to switch from high violence to low violence than the other way around. And when people switched from high violence to low or no violence, they showed low satisfaction. Similar to our findings, the reaction model [18] would predict a lower satisfaction for people who opt against violence. What our studies add to the general reactance model is that people can actually have a choice but still feel like they have none. Regret can emerge from learning the outcomes of the actions (Studies 1 and 2), but also already be anticipated before seeing outcomes (Study 4). Hypothesis 4 was supported.
5. What is the relation of choice to satisfaction in violent-interactive fiction? The enjoyment effect of high-violence choices disappears and reverses when there is no choice (Study 3). *When there is no choice*, *most people and especially females are highly dissatisfied with highly violent narratives*, see Fig 2. The results of all four studies suggest a correlation between agency and satisfaction. The attraction of choosing violence seems to be that through this choice people can increase their satisfaction by increasing their felt agency. We therefore conclude that narrative genre and perspective influence the choices of this middle group, while their choices themselves, particularly for high violence, drive satisfaction. Hypothesis 5 was only partly supported.

We had an unpredicted side finding. When choices are forced (Study 3) or appear forced (no N in Study 2 and 4), females show lower satisfaction. When all choices are available (Study 1), including no violence (N), females and males show the same satisfaction for different choice paths. This seems to suggest that women are more set on their preference, and that choice seems to play a larger role for satisfaction for females than males.

Our data suggest that while disposition may play a significant role in determining choice paths and satisfaction, there is a large middle group in the population that can be swayed to choose high violence and enjoy doing so because of this choice. That means the people in this middle group might choose either path, and the actual choice determines satisfaction. As reported, this middle group consisted of up to 66% of our participants. This number is especially surprising considering the graphic nature of violence in our stories, including in the fantasy genre, see S1 Interactive Stories. Violence-enabling and inhibiting factors (genre, perspective) influence but do not determine the choice of this group. These people already anticipate their satisfaction at the point of making a choice (Study 4) and thus can predict and thereby decide how satisfied they will be.

It is important to note that within Studies 1 and 2 respectively, the satisfaction ratings for different choice paths remained similar across different genre and person conditions, even though the distribution of choices varied significantly. Moreover, satisfaction ratings for the VV group in particular remained similarly high between Studies 1 and 2, even when more participants chose the VV path in Study 2. This distribution suggests that the actual choices people make, and not just personal disposition, matter for the satisfaction ratings. Specifically, participants in our studies mentally connected their initial choice for high violence with an opting-out of morality and opting-in for fun (Study 4). Apparently, the very act of opting for high violence simultaneously disconnects people from constraints of morality and responsibility and thereby opens an aesthetic realm of fun and satisfaction.

In our proposed framework, it is not that the large middle group enjoys violence but that they accept it in media as a route to a sense of control (agency) that is satisfying. Our studies support prior research that has found that choice itself is a driver of pleasure by allowing for a greater sense of control [14, 20, 21, 23, 37, 39] not simply disposition. Our studies show that this middle group is quite large. At the same time, this middle group was not immune to moral concerns and often defected after a first choice for high violence. This pattern of regret led to low satisfaction.

We suggest that by choosing high violence, people claim specific forms of agency over the media content, which leads to greater enjoyment. The appeal might not be the satisfaction of a disposition, but rather an act of choosing stories that break out of the ordinary and thus open up an aesthetic zone of enjoyment. Choosing violence is enjoyable, not violence itself. Reversely, people who, when given the choice, do not opt for high violence, do not claim agency, do not enter a zone of aesthetic enjoyment and do not enjoy the stories. Consequently, they stay in the realm of expected choices, feel confined by morality, experience feelings of guilt, and, despite having a choice, may not have the feeling of choice and control (agency). Our findings, if confirmed, are significant for understanding the appeal violence has for media users to satisfy a desire for aesthetic distance and heightened agency.

## Supporting information

S1 Interactive Stories All versions used in study. (DOCX)

S1 Table Given are the participant data for Study 1. In Study 1, participants had six choice paths, including a nonviolent first choice. (CSV)

S2 Table Given are the participant data for Study 2. In Study 2, participants had four choice path, excluding a first choice of non-violence. (CSV)

S3 Table Given are the participant data for Study 3. In Study 3, participants made no plot choices. (CSV)

S4 Table Given are the participant data for Study 4. In Study 4, participants rated their predicted overall satisfaction prior to making a second plot choice. (CSV)
