# Supplementary material for: Choosing and enjoying violence in narratives
Source: PLoS One. 2019 Dec 19;14(12):e0226503. doi: 10.1371/journal.pone.0226503 (PMC6922367; doi:10.1371/journal.pone.0226503)
Supplement: S1 Interactive Stories — (DOCX) [file pone.0226503.s001.docx]

**S1 Interactive Stories. All versions used in study**

*The stories are organized by genre and third or second person narrative (realistic, fantasy, nazi historical, non-nuanced historical). Each story derivative bears a number I, II or III. Each small print letter materializes a choice given to the reader. The continuation of the story that corresponds is under each capitalized letter. For instance, if I choose a, I will only read what’s happening in A.*

***Realistic Third-Person***

**I.** John is walking home from work when he sees his neighbor Ronald outside doing some yard work. Ronald often has projects going on outside that makes their whole neighborhood look like it’s under construction, which really irritates John because he really likes everything looking neat and tidy. No one else is outside. As John thinks about this, he decides to approach Ronald and

- 1. slap him.
  2. hit him with a baseball bat lying nearby.
  3. ask how his weekend is going.

A. Ronald looks at John with a confused expression and asks why John slapped him. John responds by

- 1. clapping him on the shoulder, saying he has been stressed at work lately and is sorry for taking that out on him.
  2. taking out a knife and repeatedly stabbing Ronald.

B. Ronald lies moaning on the ground looking very bruised and bloodied. John responds by then

- 1. admitting he forgot to take his medicine that morning and calling an ambulance for medical attention. He apologizes profusely and wants to make things right.
  2. taking out a gun and shooting Ronald fifteen times.

C. They chat for a few minutes and Ronald invites John over for a grill party. John then

- 1. Accepts the invite and says goodbye.
  2. Takes out a knife and repeatedly stabs Ronald.

**II.** On Edward’s way to work, he stops in Starbucks to get a morning coffee and a little breakfast. As he enters the store, he sees Jim, a coworker who he is pretty sure has been stealing his lunch. Jim, the only other person in the Starbucks, is walking out with a coffee and a bagel, and as Edward walks by him on his way to the counter,

1. He subtly hits him with his shoulder as he passes.
2. He grabs his hot coffee from Jim’s hand and throws it in his face.
3. Edward stops to say “hi” and ask Jim how his morning is going.

A. Jim, a little taken aback, looks back at Edward confusedly with his arms raised.

c. In response, Edward raises one hand and apologizes for running into him.

d. In response, Edward grabs a nearby chair and breaks it over his back.

B. Jim yelps and clutches his scalded face with his hands. As he screams,

e. Edward begins to apologize profusely, saying he doesn’t know what came over him.

f. Edward takes out his pocket knife and send its blade into Jim’s chest.

C. Edward and Jim talk for a few minutes before Edward

1. Says goodbye and continues to go get his coffee.
2. Grabs a nearby chair and break it over Jim’s back.

**III.** Anna is having trouble studying. It is the last day before her paper is due. The weather is really warm, so she has to leave her window open, even though her neighbor is mowing his lawn and thus making a lot of noise. Later that day, she goes grocery shopping, and in the pasta alley, she sees her neighbor – wearing a t-shirt, shorts, and flip flops – reaching for a jar of Bolognese. No one else is around. Feeling overwhelmed and angry at him, she

a. Slaps him.

b. Takes a jar of Bolognese and smashes it on his face.

c. Decides to calm herself and says hi to him. He talks about how this brand is the best for Bolognese and she agrees.

A. Her neighbor is shocked. He asks her why she did that. His cheek is still red from the slap. Anna explains the noise was unbearable, but he does not apologize. She then decides to

d. Say sorry and leave the store.

e. Pierce his bare foot to the blood with her high heels and leave the store.

B. The blood from the cut of the glass and the tomato sauce mix up on his ravaged face. He lies unconscious on the ground. No one seems to be coming for her, the supermarket is unusually empty this afternoon. She then decides to

f. Contact the store manager and seek help in an effort to apologize.

g. Take a piece of glass from the broken jar and cut his throat.

C. He asks her about school, and they talk for a few minutes. Then she

h. Says “have a nice day” and continues shopping.

i. Pierces his bare foot to the blood with her high heels and leave the store.

***Realistic Second-Person***

1. You are walking home from work when you see your neighbor Ronald outside doing some yard work. Ronald often has projects going on outside that makes your whole neighborhood look like it’s under construction, which really irritates you. No one else is outside. As you think about this, you decide to approach Ronald and
   1. slap him.
   2. hit him with a baseball bat lying nearby.
   3. ask how his weekend is going.

A. Ronald looks at you with a confused expression and asks why you slapped him. You respond by

- 1. clapping him on the shoulder and saying you have been stressed at work lately and are sorry for taking it out on him.
  2. taking out a knife and repeatedly stabbing Ronald.

B. Ronald lies moaning on the ground looking very bruised and bloodied. You respond by then

- 1. admitting you forgot to take your medicine that morning and calling an ambulance for medical attention. You apologize profusely and want to make things right.
  2. taking out a gun and shooting Ronald fifteen times.

C. They chat for a few minutes and Ronald invites John over for a grill party. John then

- 1. Accepts the invite and says goodbye.
  2. Takes out a knife and repeatedly stabs Ronald.

**II.** On your way to work, you stop in Starbucks to get a morning coffee and a little breakfast. As you enter the store, you see Jim, a coworker who you are pretty sure has been stealing your lunch. Jim, the only other person in the Starbucks, is walking out with a coffee and a bagel, and as you walk by him on your way to the counter,

1. You subtly hit him with your shoulder as you pass.
2. You grab his hot coffee from his hand and throw it in his face.
3. You stop to say “hi” and ask how his morning is going.

A. Jim, a little taken aback, looks back at you confusedly with his arms raised.

d. In response, you raise one hand and apologize for running into him.

e. In response, you grab a nearby chair and break it over his back.

B. Jim yelps and clutches his scalded face with his hands. As he screams,

f. You begin to apologize profusely, saying you don’t know what came over you.

g. You take out your pocket knife and send its blade into Jim’s chest.

C. You talk for a few minutes before you

1. Say goodbye and continue to go get your coffee.
2. Grab a nearby chair and break it over Jim’s back.

**III.** You are having trouble studying. It is the last day before your paper is due. The weather is really warm, so you have to leave the window open, even though your neighbor is mowing his lawn and thus making a lot of noise. Later that day, you go grocery shopping, and in the pasta alley, you see your neighbor – wearing a t-shirt, shorts, and flip flops – reaching for a jar of Bolognese. No one else is around. Feeling overwhelmed and angry at him, you

a. Slap him

b. Take a jar of Bolognese and smash it on his face.

c. decide to calm yourself and say hi to him, he talks about how this brand is the best for Bolognese and you agree.

A. Your neighbor is shocked. He asks you why you did that. His cheek is still red from the slap. You explain the noise was unbearable, but he does not apologize. You then decide to

d. Say sorry and leave the store.

e. Pierce his bare foot to the blood with your heels and leave.

B. The blood from the cut of the glass and the tomato sauce mix up on his ravaged face. He lies unconscious on the ground. No one seems to be coming for you, the supermarket is unusually empty this afternoon. You then decide to

f. Contact the store manager to seek help in an effort to apologize.

g. Take a piece of glass from the broken jar and cut his throat.

C. He asks you about school, and you talk for a few minutes. Then you

h. Say: “have a nice day” and continue shopping.

i. Pierce his bare foot to the blood with your heels and leave the store.

***Fantasy Third-Person***

1. The great giant Griz is walking home through the enchanted forest when he sees his neighbor, a large troll named Ronald, outside collecting some water nymphs to take home. Ronald often collects noisy creatures like sprites, tree elves, nymphs, and miniature unicorns that make their whole section of the forest look like it’s a mystical zoo, which really irritates Griz because he likes everything looking neat and tidy. No one else is in the woods. As Griz thinks about this, he decides to approach Ronald and
   1. slap him.
   2. hit him with a large, thorny branch lying nearby.
   3. ask how his weekend is going.

A. Ronald looks at Griz with a confused expression and asks why Griz slapped him. Griz responds by

- 1. clapping him on the shoulder, saying he has been stressed about insurgent fairies lately and is sorry for taking that out on him.
  2. taking out a poisoned knife a witch gave him and repeatedly stabbing Ronald.

B. Ronald lies moaning on the ground looking very bruised and bloodied. Griz responds by then

- 1. admitting he forgot to drink his potion that morning and calling a wizard for medical attention. He apologizes profusely and wants to make things right.
  2. taking out a bow and shooting Ronald fifteen times with magical, poisonous arrows.

C. They chat for a few minutes and Ronald invites Griz over for a festival in the woods. He then

- 1. accepts the invite and says goodbye.
  2. takes out a poisoned knife a witch gave him and repeatedly stabs Ronald.

**II.** On Sir Edward’s way to Griamore castle, he stops at the Celestial Tree to harvest some of its volatile Stardust. All is quiet in the land around him, and he believes himself alone until he sees Sir John walking toward the tree, one of his fellow knights who he believes has been stealing his ale and potions. With a sack of his newly-harvested stardust in hand, Sir Edward walks up to Sir John

1. And subtly hits him with his shoulder as he passes.
2. And throws his stardust into his face where it begins to burn Sir John’s skin.
3. And say “hi” and ask how his day is going.
4. Sir John, a little taken aback, looks back at Sir Edward confusedly with his arms raised.

d. In response, Sir Edward raises one hand and apologizes for running into him.

e. In response, Sir Edward grabs a nearby log and breaks it over his back.

B. Sir John yelps and clutches his sizzling face with his hands. As he screams,

f. Sir Edward begins to apologize profusely, saying he doesn’t know what came over him.

g. Sir Edward grabs his cursed dagger and sends its blade into Sir John’s chest.

C. Sir Edward and Sir John talk for a few minutes before Sir Edward

h. Says goodbye and continues on his way.

i. Grabs a nearby log and breaks it over Sir John’s back.

**III.** Once upon a time, in an enchanted forest, lived the beautiful maiden Snow White. She is sharing her house with the seven dwarfs. She is having trouble reading and really wants to finish her book. It is really warm, so she has to leave her window open, but the dwarfs are playing very loudly with each other. Later that day, she goes to the market to get some food, and at one of the stands, she sees one of the dwarfs – wearing his cap – reaching for a jar of applesauce. Feeling overwhelmed with anger at the remembrance of the noise and the infamous poisoned apple from her past, she decides to

a. Slap him.

b. Take a jar of applesauce and smash it on his face.

c. Be nice and gentle and start talking about the weather.

A. The dwarf is shocked and he asks her why she did that. His cheek is still red from the slap. Snow White explains that the noise was unbearable, but he does not apologize. She then decides to

d. Say sorry and leave the market

e. Pierce his hairy barefoot to the blood with her heels and leave.

B. The blood from the cut of the glass and the applesauce mixes up on his ravaged face and his little bonnet. He lays unconscious on the ground. No one seems to be coming for her, the market is unusually empty this afternoon. She then decides to

f. Seek for help in an attempt to apologize for her actions.

g. Take a piece of glass from the broken jar and cut his throat.

C. The dwarf smiles at her and comments on the weather. She helps him reach for the jar. Then she

h. Says goodbye and leaves gracefully

i. Pierces his hairy barefoot to the blood with her heels and leave.

***Fantasy Second-Person***

1. You are a great giant and are walking home through the enchanted forest when you sees your neighbor, a large troll named Ronald, outside collecting some water nymphs to take home. Ronald often collects noisy creatures like sprites, tree elves, nymphs, and miniature unicorns that make your whole section of the forest look like it’s a mystical zoo, which really irritates you because you like everything looking neat and tidy. No one else is in the woods. As you think about this, you decide to approach Ronald and
   1. slap him.
   2. hit him with a large, thorny branch lying nearby.
   3. Ask him how his weekend is going.

A. Ronald looks at you with a confused expression and asks why you slapped him. You respond by

- 1. clapping him on the shoulder, saying you have been stressed about insurgent fairies lately and are sorry for taking that out on him.
  2. taking out a poisoned knife a witch gave him and repeatedly stabbing Ronald.

B. Ronald lies moaning on the ground looking very bruised and bloodied. You respond by then

- 1. admitting you forgot to drink your potion that morning and calling a wizard for medical attention. You apologize profusely and want to make things right.
  2. taking out a bow and shooting Ronald fifteen times with magical, poisonous arrows.

C. You chat for a few minutes and Ronald invites you over for a festival in the woods. You then

- 1. accept the invite and say goodbye.
  2. take out a poisoned knife a witch gave him and repeatedly stabs Ronald.

1. On your way to Griamore castle, you stop at the Celestial Tree to harvest some of its volatile Stardust. All is quiet in the land around you, and you believe yourself alone until you see Sir John walking toward the tree, one of your fellow knights who you believe has been stealing your ale and potions. With a sack of your newly-harvested stardust in hand, you walk up to Sir John
2. And subtly hit him with your shoulder as you pass.
3. And throw your stardust into his face where it begins to burn his skin.
4. And say “hi” and ask how his day is going.
5. Sir John, a little taken aback, looks back at you confusedly with his arms raised.

d. In response, you raise one hand and apologize for running into him.

e. In response, you grab a nearby log and break it over his back.

B. Sir John yelps and clutches his sizzling face with his hands. As he screams,

f. you begin to apologize profusely, saying you don’t know what came over you.

g. you grab your cursed dagger and send its blade into Sir John’s chest.

C. You talk for a few minutes before you

h. Say goodbye and continue on your way.

i. Grab a nearby log and break it over Sir John’s back.

**III.** Once upon a time, in an enchanted forest, there lived with seven lively dwarfs. You go there to live with them to hide from an evil queen. One day, you are having trouble reading and really want to finish your book. It is really warm, so you have to leave your window open, but the dwarfs are playing very loudly with each other. Later that day, you go to the market to get some food, and at one of the stands, you see one of the dwarfs – wearing his cap – reaching for a jar of applesauce. Feeling overwhelmed with anger at the remembrance of the noise and a poisoned apple, you decide to

a. Slap him.

b. Take a jar of applesauce and smash it on his face.

c. Be nice and gentle and start talking about the weather

A. The dwarf is shocked and he asks you why you did that. His cheek is still red from the slap. You explain that the noise was unbearable, but he does not apologize. You then decide to

d. Say sorry and leave.

e. Pierce his hairy bare foot to the blood with your heels and leave.

B. The blood from the cut of the glass and the applesauce mix upon his ravaged face and his little bonnet. He lies unconscious on the ground. No one seems to be coming for you, the market is huge and empty this afternoon. You then decide to

f. Seek for help in an attempt to apologize for your actions.

g. Take a piece of glass from the broken jar and cut his throat.

C. The dwarf smiles at you and comments on the weather. You help him reach for the jar. Then you

h. Say goodbye and leave gracefully

i. Pierce his hairy barefoot to the blood with your heels and leave.

***Nazi-Historical Third-Person***

1. It’s 1944 and World War II is still raging. Johannes is a German Nazi and is walking home from work when he sees his neighbor Ronald outside doing some yard work. Ronald often has projects going on outside that makes the whole neighborhood look like it’s under construction, which really irritates Johannes. No one else is outside. As Johannes thinks about this, he decides to approach Ronald and
   1. slap him.
   2. hit him with a baseball bat lying nearby.
   3. ask how his weekend is going.

A. Ronald looks at Johannes with a confused expression and asks why he slapped him. Johannes responds by

- 1. clapping him on the shoulder and saying he has been stressed with work assignments lately and is sorry for taking it out on him.
  2. taking out a knife and repeatedly stabbing Ronald.

B. Ronald lies moaning on the ground looking very bruised and bloodied. Johannes responds by then

- 1. admitting he wasn’t thinking straight and running to find a doctor for medical attention. He apologizes profusely and wants to make things right.
  2. taking out a gun and shooting Ronald fifteen times.

C. They chat for a few minutes and Ronald invites him over for a beer later. Johannes then

- 1. accepts the invite and says goodbye.
  2. takes out a knife and repeatedly stabs Ronald.

1. It is 1938 in Berlin and Edward is a low-ranking member of the Nazi party. On his way to work, he stops in the local cafe to get a morning coffee and a little breakfast. As Edward enters the store, he sees Jurgen, a coworker who he is pretty sure has been stealing his lunch. Jurgen, the only other person in the little store, is walking out with a coffee, and as Edward walks by him on his way to the counter,
2. He subtly hits Jurgen with his shoulder as he passes.
3. He grabs Jurgen’s hot coffee from his hand and throws it in his face.
4. He says “hi” and asks him how his morning is going.

A. Jurgen, a little taken aback, looks back at Edward confusedly with his arms raised.

d. In response, Edward raises one hand and apologizes for running into him.

e. In response, Edward grabs a nearby chair and breaks it over his back.

B. Jurgen yelps and clutches his scalded face with his hands. As Jurgen screams,

f. Edward begins to apologize profusely, saying he doesn’t know what came over him.

g. Edward takes out his pocket knife and sends its blade into Jurgen’s chest.

C. Edward and Jugen talk for a few minutes before Edward

h. Says goodbye and continues to go get his coffee.

i. Grabs a nearby chair and breaks it over Jurgen’s back.

**III.** It is Summer 1940. Second World War is at its peak in Germany, and Anna is a Nazi living in Berlin. She is having trouble reading because of some noise outside. She really wants to finish the morning newspaper. It is really warm, so she has to leave the window open, and her neighbor keeps playing the radio, making a lot of noise. Later, Anna goes to the shop and sees her neighbor asking for a jar of milk. No one else is around. Feeling overwhelmed and angry with him, Anna

a. Slaps him.

b. Takes the jar of milk and smashes it on his face.

c. Asks him what he thinks about the recent progress of the war

A. Anna’s neighbor is shocked and asks her why she did that. His cheek is still red from the slap. She explains the noise was unbearable but he does not apologize. Anna then decides to

d. Say sorry and leave.

e. Pierce his foot with her heels until it bleeds and then leave.

B. The blood from his cuts and the milk from the jar mix on his ravaged face. He lays unconscious on the ground. No one seems to be coming for Anna, and the store is empty. She then decides to

f. Help him by taking a piece of cloth and soothing his wounds in an attempt to apologize.

g. Take a piece of glass from the broken jar and cut his throat.

C. Anna’s neighbor replies and they chat for a few minutes. Anna then

h. Wishes him a nice day and leave

i. Pierces his foot with your boot heels until it bleeds and then leaves.

***Nazi-Historical Second-Person***

1. It’s 1944 and World War II is still raging. You are a German Nazi and are walking home from work when you see your neighbor Ronald outside doing some yard work. Ronald often has projects going on outside that makes your whole neighborhood look like it’s under construction, which really irritates you. No one else is outside. As you think about this, you decide to approach Ronald and
   1. slap him.
   2. hit him with a baseball bat lying nearby
   3. ask how his weekend is going.

A. Ronald looks at you with a confused expression and asks why you slapped him. You respond by

- 1. clapping him on the shoulder and saying you have been stressed with work assignments lately and are sorry for taking it out on him.
  2. taking out a knife and repeatedly stabbing Ronald.

B. Ronald lies moaning on the ground looking very bruised and bloodied. You respond by then

- 1. admitting you weren’t thinking straight and running to find a doctor for medical attention. You apologize profusely and want to make things right.
  2. taking out a gun and shooting Ronald fifteen times.

C. You chat for a few minutes and Ronald invites you over for a beer later. You then

- 1. accept the invite and say goodbye.
  2. Take out a knife and repeatedly stab Ronald.

1. It is 1938 in Berlin and you are a low-ranking member of the Nazi party. On your way to work, you stop in the local cafe to get a morning coffee and a little breakfast. As you enter the store, you see Jurgen, a coworker who you are pretty sure has been stealing your lunch. Jurgen, the only other person in the little store, is walking out with a coffee, and as you walk by him on your way to the counter,
   1. You subtly hit him with your shoulder as you pass.
   2. You grab his hot coffee from his hand and throw it in his face.
   3. You say “hi” and ask him how his morning it going.

A. Jurgen, a little taken aback, looks back at you confusedly with his arms raised.

d. In response, you raise one hand and apologize for running into him.

e. In response, you grab a nearby chair and break it over his back.

B. Jurgen yelps and clutches his scalded face with his hands. As he screams,

f. You begin to apologize profusely, saying you don’t know what came over you.

g. You take out your pocket knife and send its blade into Jurgen’s chest.

C. You talk for a few minutes before you

h. Say goodbye and continue to go get your coffee.

- - 1. Grab a nearby chair and break it over Jurgen’s back.

**III.** It is Summer 1940. Second World War is at its peak in Germany, and you are a Nazi living in Munich. You are having trouble reading because of some noise outside. You really want to finish the morning newspaper. It is really warm, so you have to leave the window open, and your neighbor keeps playing the radio, making a lot of noise. Later, you go to the shop and see your neighbor reaching for a jar of milk. No one else is around. Feeling overwhelmed and angry with him, you

a. Slap him.

b. Take the jar of milk and smash it on his face.

c. Ask him what he thinks about the recent progress of the war

A. Your neighbor is shocked and asks you why you did that. His cheek is still red from the slap. You explain the noise was unbearable but he does not apologize. You then decide to

d. Say sorry and leave.

e. Pierce his foot with your boot heels until it bleeds and then leave.

B. The blood from his cuts and the milk from the jar mix on his ravaged face. He lays unconscious on the ground. No one seems to be coming for you, and the store is empty. You then decide to

f. Help him by taking a piece of cloth and soothing his wounds in an attempt to apologize.

g. Take a piece of glass from the broken jar and cut his throat.

C. Your neighbor replies and you start chatting for a few minutes. You then

h. Wish him a nice day and leave.

i. Pierce his foot with your boot heels until it bleeds and then leave.

***General-Historical Third Person***

1. It’s 1900 in New York City. John is walking home from his factory job when he sees his neighbor Ronald outside pruning his bushes. Ronald often has projects going on outside that makes the whole neighborhood look like it’s under construction, which really irritates John. No one else is outside. As John thinks about this, he decides to approach Ronald and
   1. slap him.
   2. hit him with a baseball bat lying nearby.
   3. ask how his weekend is going.

A. Ronald looks at John with a confused expression and asks why he slapped him. John responds by

- 1. clapping him on the shoulder and saying he has been stressed with long work hours lately and is sorry for taking it out on him.
  2. taking out a knife and repeatedly stabbing Ronald.

B. Ronald lies moaning on the ground looking very bruised and bloodied. John responds by then

- 1. admitting he wasn’t thinking straight and running to find a doctor for medical attention. He apologizes profusely and wants to make things right.
  2. taking out a gun and shooting Ronald fifteen times.

C. They chat for a few minutes and Ronald invites him over for a beer later. John then

- 1. accepts the invite and says goodbye.
  2. takes out a knife and repeatedly stabs Ronald.

1. It is 1714 in London and Mr. Richardson works in a local printery. On his way to work, he stops in the local cafe to get a morning tea and a little breakfast. As Mr. Richardson enters the store, he sees Mr. Pepys, a fellow printer who he is pretty sure has been stealing his lunch. Mr. Pepys, the only other person in the little store, is drinking a tea, and as Mr. Richardson walks by him on his way to the counter,
   1. He subtly hits Mr. Pepys with his shoulder as he passes.
   2. He grabs Mr. Pepys’s hot tea from his hand and throws it in his face.
   3. He stops to say “hi” and ask him how his morning is going.

A. Mr. Pepys, a little taken aback, looks back at Mr. Richardson confusedly with his arms raised.

d. In response, Mr. Richardson raises one hand and apologizes for running into him.

e. In response, Mr. Richardson grabs a nearby chair and breaks it over his back.

B. Mr. Pepys yelps and clutches his scalded face with his hands. As he screams,

f. Mr. Richardson begins to apologize profusely, saying he doesn’t know what came over him.

g. Mr. Richardson takes out his pocket knife and sends its blade into Mr. Pepys’s chest.

C. Mr. Richardson and Mr. Pepys talk for a few minutes before Mr. Richardson

h. Says goodbye and continues to go get his tea.

i. Grabs a nearby chair and breaks it over Mr. Pepys’s back.

**III.** It is 1853 and Anna lives in Victorian London. She is having trouble reading because of some noise outside. She really wants to finish the morning newspaper. It is really warm, so she has to leave the window open, and her neighbor keeps playing the piano, making a lot of noise. Later, Anna goes to the market and sees her neighbor reaching for a jar of milk. No one else is around. Feeling overwhelmed and angry with him, Anna

a. Slaps him.

b. Takes the jar of milk and smashes it on his face.

c. Starts talking about the weather with her neighbor and how hot it has been recently

A. Anna’s neighbor is shocked and asks her why she did that. His cheek is still red from the slap. She explains the noise was unbearable but he does not apologize. Anna then decides to

d. Say sorry and leave.

e. Pierce his foot with her heels until it bleeds and then leave.

B. The blood from his cuts and the milk from the jar mix on his ravaged face. He lays unconscious on the ground. No one seems to be coming for Anna, and the store is empty. She then decides to

f. Help him by taking a piece of cloth and soothing his wounds in an attempt to apologize.

g. Take a piece of glass from the broken jar and cut his throat.

C. Anna’s neighbor also complains about the heat. She then

h. Wishes him a nice day and leave

i. Pierces his foot with her boot heels until it bleeds and then leaves.

***General Historical Second-Person***

1. It’s 1900 in New York City. You are walking home from your factory job when you see your neighbor Ronald outside pruning his bushes. Ronald often has projects going on outside that makes the whole neighborhood look like it’s under construction, which really irritates you. No one else is outside. As you think about this, you decide to approach Ronald and
   1. slap him.
   2. hit him with a baseball bat lying nearby.
   3. ask how his weekend is going.

A. Ronald looks at you with a confused expression and asks why you slapped him. You respond by

- 1. clapping him on the shoulder and saying you have been stressed with long work hours lately and are sorry for taking it out on him.
  2. taking out a knife and repeatedly stabbing Ronald.

B. Ronald lies moaning on the ground looking very bruised and bloodied. You respond by then

- 1. admitting you weren’t thinking straight and running to find a doctor for medical attention. You apologize profusely and want to make things right.
  2. taking out a gun and shooting Ronald fifteen times.

C. You chat for a few minutes and Ronald invites you over for a beer later. You then

- 1. accept the invite and say goodbye.
  2. take out a knife and repeatedly stab Ronald.

1. It is 1714 in London and you work in a local printery. On your way to work, you stop in the local cafe to get a morning tea and a little breakfast. As you enter the store, you see Mr. Pepys, a fellow printer who you are pretty sure has been stealing your lunch. Mr. Pepys, the only other person in the little store, is drinking a tea, and as you walk by him on your way to the counter,
   1. You subtly hit him with your shoulder as you pass.
   2. You grab his hot tea from his hand and throw it in his face.
   3. You stop to say “hi” and ask him how his morning is going.

A. Mr. Pepys, a little taken aback, looks back at you confusedly with his arms raised.

d. In response, you raise one hand and apologize for running into him.

e. In response, you grab a nearby chair and break it over his back.

B. Mr. Pepys yelps and clutches his scalded face with his hands. As he screams,

f. You begin to apologize profusely, saying you don’t know what came over you.

g. You take out your pocket knife and send its blade into Mr. Pepys’s chest.

C. You talk for a few minutes before you

h. Say goodbye and continue to go get your tea.

1. Grab a nearby chair and break it over Mr. Pepys’s back.

**III.** It is 1853 and you live in Victorian London. You are having trouble reading because of some noise outside. You really want to finish the morning newspaper. It is really warm, so you have to leave the window open, and your neighbor keeps playing the piano, making a lot of noise. Later, you go to the market and see your neighbor reaching for a jar of milk. No one else is around. Feeling overwhelmed and angry with him, you

a. Slap him.

b. Take the jar of milk and smash it on his face.

c. Start talking about the weather and how hot it has been recently

A. Your neighbor is shocked and asks you why you did that. His cheek is still red from the slap. You explain the noise was unbearable but he does not apologize. You then decide to

d. Say sorry and leave.

e. Pierce his foot with the heel of your shoe until it bleeds and then leave.

B. The blood from his cuts and the milk from the jar mix on his ravaged face. He lays unconscious on the ground. No one seems to be coming for you, and the market is empty. You then decide to

f. Help him by taking a piece of cloth and soothing his wounds in an attempt to apologize.

g. Take a piece of glass from the broken jar and cut his throat.

C. Your neighbor complains about the heat too and you

h. Wish him to have a nice day and leave

i. Pierce his foot with the heel of your shoe until it bleeds and then leave.
